# Psychological Construction and Cultivation of New Entrepreneurs Using Industrial Cluster Theory and Multidimensional Structure Model

**DOI:** 10.3389/fpsyg.2021.693377

**Published:** 2021-08-25

**Authors:** Huiling Fan

**Affiliations:** School of Economics and Trade, Henan University of Technology, Zhengzhou, China

**Keywords:** industrial cluster theory, multidimensional structure model, new entrepreneurs, psychological characteristics, entrepreneurial value

## Abstract

This study aims to analyze the influencing factors and training plans of cultivating new entrepreneurs in the new era based on industrial cluster theory. First, the status of the research on the correlation between psychological characteristics and entrepreneurial performance of new entrepreneurs is reviewed. Second, 200 new entrepreneurs in the Xi’an City of Shaanxi Province are randomly selected as the research objects. An empirical model of the correlation between entrepreneurial psychological characteristics and entrepreneurial performance is innovatively proposed. Finally, the questionnaire data are analyzed by correlation analysis and SPSS 26.0 (Chicago) statistical analysis. The results show that most of the entrepreneurs are between 30 and 50 years old, with a larger number of mens, and most graduate from junior colleges. The gender and education level of the new entrepreneurs have a little impact on their entrepreneurial performance (*P* > 0.05), whereas their age has a significant impact on their entrepreneurial performance (*P* < 0.05). Moreover, there is a significant correlation between the psychological characteristics of the entrepreneur and the two dimensions of entrepreneurial performance (development and profitability) (*P* < 0.05). This shows that the key to cultivating new entrepreneurs is to develop their age and psychological characteristics, which can enhance the entrepreneurial ability of new entrepreneurs and provide a basis for cultivating multidimensional entrepreneurial talents in new enterprises.

## Introduction

An industrial cluster means that enterprises or factories share some production resources through geographical aggregation. Some scholars believe that the competition and cooperation in the same industry and on the social network are based on trust, sharing professional labor, resources, and knowledge. Which countries should be chosen is a matter that China needs to be fully considered (Qu et al., 2017). Cluster planning becomes a method of formulating development policies by the government. The United Kingdom, the United States, the European Union, and other developed countries promote the development of national innovation industries by establishing high-tech zones, and their successful experience is quickly imitated by developing countries (Mayhew et al., 2016).

Although it has a positive development trend, the industrial cluster of China still has some problems. Zhang and Hu (2017) argued that the lack of innovation mechanisms and the construction of an entrepreneurial environment are still problems that cannot be ignored in the industrial clusters of China. Most of the industrial clusters lack innovative ability, and the gap between the advanced industrial clusters of the world is great (Chen and Xin, 2019). Iodice et al. (2017) found that the ability of research and development and the transformation rate of scientific and technological achievements of the industrial cluster of China should be improved. In addition, Park (2017) mentioned that innovation ability, cluster location selection, cluster subject relationship, and interaction intensity, and the availability of venture capital have a major impact on the transformation efficiency of scientific and technological achievements in China. These factors need to be further analyzed and given more attention to the industrial parks of China (Iodice et al., 2017; Park, 2017). Compared with the developed industrial clusters in the world, the industrial clusters of China still have a long way to go, because the competitiveness, especially the international competitive ability, is still weak (Redondo, 2019).

The primary problem to be solved in improving the ability of industrial clusters of China is to construct an entrepreneurial environment. Entrepreneurs, as the promoters and implementers of constructing an entrepreneurial environment, play an important role in entrepreneurial activities, and they can more effectively identify opportunities, allocate resources, think about strategies, and ultimately create new businesses. In this case, start-ups not only achieve economic development but also realize their goals and give full play to their potentials (Yuan et al., 2020). After the studies on entrepreneurship are reviewed, it is found that most of them are about the entrepreneurial phenomenon, entrepreneurial behavior, entrepreneurial environment, or the induction of foreign entrepreneurial research. The discussions on the relationship between the demographic characteristics of entrepreneurs and the natural characteristics of entrepreneurs are inadequate (Hashai and Markovich, 2017; Shen et al., 2019).

In short, there are some problems in industrial clusters of China, such as the lack of innovation mechanism, the low transformation rate of scientific research results, the lack of international competitiveness, and the lack of research on the relationship between demographic characteristics of entrepreneurs and the natural characteristics of entrepreneurs. Therefore, based on the characteristics of industrial clusters in the new era, a multidimensional structure empirical model of the correlation between entrepreneurial psychological characteristics and entrepreneurial performance is put forward, and it is also the innovation point of the study. The influencing factors of the psychological construction of new entrepreneurs are analyzed, providing the basis for improving the efficiency of new enterprises.

## Materials and Methods

### Industrial Cluster Theory From the Perspective of Agglomeration in the New Era

Cluster planning can improve the transformation rate of scientific and technological achievements. However, the industrial cluster ability of China is relatively weak, as mentioned in the introduction part of the article. Therefore, the industrial cluster theory in the new era is analyzed, and this provides a theoretical basis for cultivating new entrepreneurs for enterprises.

An industrial cluster refers to a group of enterprises, professional suppliers, service providers, financial institutions, manufacturers of related industries, and other related institutions that are geographically concentrated in a specific region and have competitive and cooperative relations. The development level of the industrial cluster becomes an important investigation indicator of a region and an economic union (Panda and Nagendra, 2017; Yang and Dunford, 2017), as shown in Table 1.

**TABLE 1:** Characteristics of industrial clusters in the new era.

Feature classification	Content
Close correlation	Most enterprises in each geographical area focus on the same industry or closely related industries
Specialization	There are certain or several significant industrial characteristics among enterprises within the same industry
Supply and demand connectivity	The supply and demand of the whole cluster are linked to realize local purchases, showing the advantage of the cluster members
Market share	The whole cluster has a significant market share
Market penetration	In the process of development, some clusters advocate interactive development between industrial clusters and regional professional markets
Learning effect	A successful enterprise often provides other enterprises in the industrial cluster with labor division and cooperation

### Research Status of the Correlation Between the Psychological Characteristics of New Entrepreneurs and the Performance of New Ventures

The characteristics are the personality of an individual, and it is formed by a variety of rational characteristics under the interaction of heredity and environment (Link and Lashley, 2016). Performance refers to achieving a predetermined goal or completing a specific task, and it is usually functional or effective. The purpose of all business operations is to improve the performance of enterprises. Entrepreneurial performance is usually used as the indicator of organizational activities to evaluate the operation of new enterprises. Thus, entrepreneurial performance is the main reference standard to measure the success of entrepreneurial activities (Or et al., 2015; Przepiorka, 2016).

The potential characteristics of entrepreneurs are constantly shown in entrepreneurial practice. Entrepreneurs give full play to their characteristics, figure out the entrepreneurial opportunities in a complex environment, and take full advantage of entrepreneurial resources to establish new enterprises successfully and achieve economic and social profits (Ratna, 2018). Some researchers propose that the dependence of entrepreneurs on resources and personal characteristics has a great impact on the success of enterprises, and it also plays an important role in promoting the development and growth of science and technology (Heinz et al., 2017; Lv et al., 2021).

The current studies have a remarkable disconnect between psychological characteristics and entrepreneurship. Wu et al. (2019) studied the relationship between a certain dimension of psychological capital of an entrepreneur (such as entrepreneurial self-efficacy) and the performance of new ventures and pointed out that entrepreneurial self-efficacy plays a mediating role in the positive impact of entrepreneurial education behavior. Nevertheless, the number of studies is small, and the conclusions are inconsistent. Besides, the existing entrepreneurship research focuses on the impact of entrepreneurial personality, entrepreneur human capital and social capital, entrepreneurial environment and entrepreneurial strategy, and the performance of new enterprises, ignoring the role of entrepreneurial psychological characteristics (Ogas et al., 2019).

### Establishment of Research Model and Hypotheses

1. Based on the review of entrepreneurship research, some psychological characteristics for entrepreneurial success are summarized (Labrador et al., 2016; Mooradian et al., 2016), and the entrepreneurial psychological characteristics are analyzed from the five perspectives, namely success motivation, tolerance, risk bias, control tendency, and self-confidence, as shown in Table 2.

**TABLE 2:** Five psychological characteristics.

Psychological characteristics	Main content
Success motivation	The individual is committed to his/her work and wants it to achieve the best. Wu and Wu (2019) concluded that employees will engage in their work if they think it is valuable, which plays an intermediary role in the positive impact of innovative behavior, showing that the cultivation of innovative entrepreneurial talents should consider whether the entrepreneurs think the job is valuable (Wu and Wu, 2019)
Tolerance	For something uncertain, tolerate emergency and ambiguity are taken in the process of consultation
Risk bias	It refers to the attitude toward the risks (Chen, 2019)
Control tendency	It refers to the degree to which one controls his/her destiny (Felice et al., 2019)
Self-confidence	It is a sense of self-efficacy and reflects confidence of an individual to complete an activity (Rogoza et al., 2018)

**FIGURE 1:**
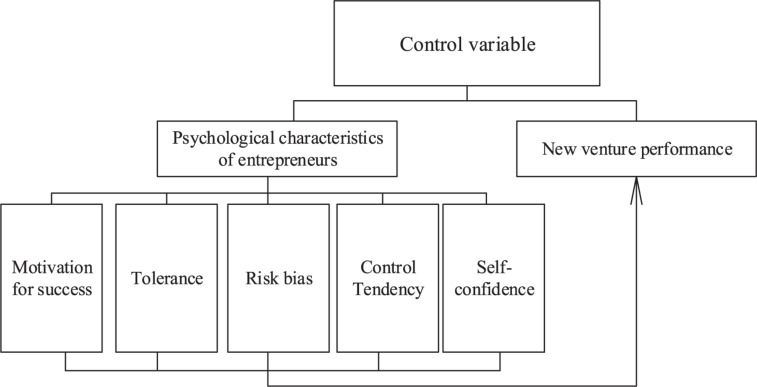
Multidimensional structure model.

The empirical model established in the study is shown in Figure 1.

2. Two dimensions of entrepreneurial performance are selected to analyze the dimensions, and they are development (Friedman et al., 2016) and profitability (Chen et al., 2017). The growth indicators can reflect the entrepreneurial process of enterprises. The above two dimensions are scored by the Likert 5-point system. The entrepreneurial performance is shown in Table 3 and includes two dimensions, namely development and profitability. Each dimension has four options numbered A, B, C, and D. The development dimension tells the growth of entrepreneurs, the development speed of enterprises, the increase of the number of employees, and the mobility of employees, whereas the profitability shows the profit of enterprises, capital turnover, and capital linkage and benefit growth.
3. The research hypotheses are brought up. The essential characteristics of entrepreneurs are gradually strengthened in the continuous entrepreneurial practice. The personality embodied in the theoretical characteristics enables entrepreneurs to identify entrepreneurial opportunities in a complex environment, make full use of entrepreneurial resources, and successfully set up innovative enterprises (Wu and Song, 2019). Moreover, entrepreneurial performance is also reflected in the entrepreneurial process. Based on the analysis, the following question is asked: Will entrepreneurial characteristics affect entrepreneurial performance?

**TABLE 3:** Entrepreneurial performance measurement.

Entrepreneurial performance		
Dimension	Development	Profitability
Options	It grows a lot in the process of enterprise establishment Compared with other enterprises in the industry, it develops very rapidly Compared with other enterprises in the industry, personnel in the enterprise increase by a large number Compared with other enterprises in the industry, the personnel mobility of the enterprise is low	The profit obtained is significant The capital transfer is fast Compared with other enterprises in the industry, the capital chain is more stable Compared with other enterprises in the industry, the efficiency is improving faster

The corresponding hypotheses are proposed:

H1: The psychological characteristics of new entrepreneurs have an impact on the development of the enterprise.
H2: The psychological characteristics of new entrepreneurs have an impact on the profitability of the enterprise.

4. The questionnaire is designed, and the scales are designed according to five main psychological characteristics, as shown in Table 4.

**TABLE 4:** Scale design.

Scale type	Design content
Success motivation	The scale is designed according to the self-oriented achievement motivation scale [30] developed by Yu Anbang et al. and Likert 5-point scale. The options are listed from “disagree” to “totally agree,” and the corresponding score is 1–5 points in turn. The higher the score is, the stronger the motivation for success is. The motivation for success is determined by the individual
Tolerance scale	The relevant scales developed by S. Bunder are used. Six typical questions are selected, and three of them are scored in reverse. The Likert 5-point is still used, and the corresponding scores from “disagree” to “totally agree” are 1–5 points in turn. The entrepreneurs interviewed pick up one of the five options. The higher the score is, the lower the tolerance of uncertainty of the entrepreneur is. The lower the score is, the higher the tolerance of uncertainty of the entrepreneur is
Risk bias scale	According to the “entrepreneur characteristics questionnaire” (Torres-Coronas and Vidal-Blasco, 2016), specific measurement indicators are used. The higher the total score is, the more risks the entrepreneur likes to take and the more obvious his/her risk bias is
Control disposition scale	Based on the Spectord’s scale, five positive questions are designed. The Likert 5-point scale is used and the corresponding scores from “disagree” to “totally agree” are 1–5 points in turn. The higher the total score is, the better the entrepreneur controls their internal personality
Self-confidence scale	Based on the self-confidence scale proposed by M. Rosenberg, five positive tests are selected to evaluate the quality of personal self-perception. The higher the score is, the more confident entrepreneurs are in the entrepreneurial process

### Analysis Tools

1. Confirmatory factor analysis is used to test the validity of the scale, and the Cronbach’s α value tests the reliability.
2. Hypotheses H1 and H2 are verified by correlation analysis. Correlation analysis explores whether there is a correlation between two variables. As the scores of psychological characteristics and entrepreneurial performance are continuous variables, their correlation is verified by Pearson’s difference product correlation method. The higher the absolute value of Pearson’s correlation coefficient, the stronger the correlation between the two variables is, and vice versa. The *t*-test is used to analyze the influence of the gender of the entrepreneur on different psychological characteristics, and one-way ANOVA is used to test the influence of the age and education level of the entrepreneur on different psychological characteristics.
3. SPSS 26.0 statistical software is used for analyzing the collected questionnaire data and Origin 2018Bit for visualizing the data.

## Results and Analysis

### Reliability and Validity of the Questionnaire

**FIGURE 2:**
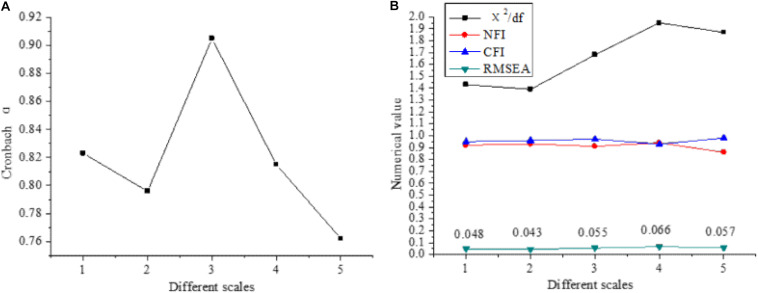
Reliability and validity analysis of the questionnaire [**(A)** reliability; **(B)** validity; 1: success motivation scale; 2: tolerance scale; 3: risk bias scale; 4: control tendency scale; 5: self-confidence scale].

The reliability and validity of the questionnaire are analyzed, and the results are shown in Figures 2A,B.

Figure 2 shows that the Cronbach’s α of each scale is between 0.76 and 0.92, χ^2^/df is less than 2, and MSEA is more than 0.05. The results show that the reliability of the scale is high and has a good fitting ability.

### Descriptive Statistics of the Personal Information of New Entrepreneurs

**FIGURE 3:**
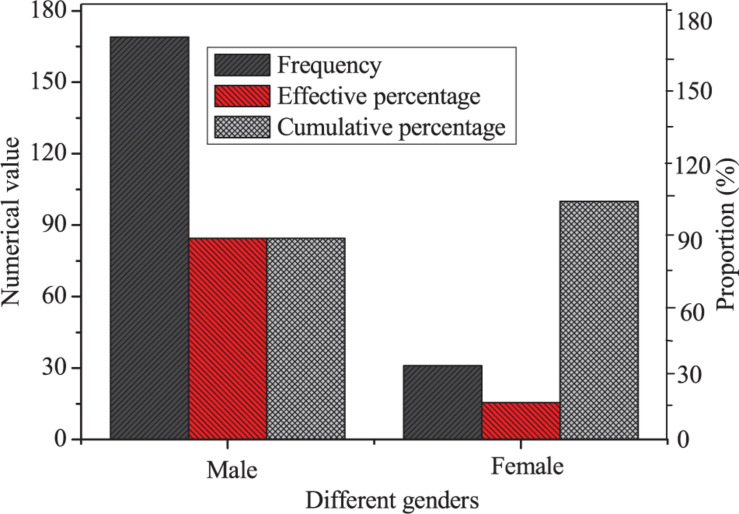
Descriptive statistics of the genders of entrepreneurs.

First, descriptive statistics of the genders of entrepreneurs are made, and the results are shown in Figure 3.

Figure 3 indicates that the gender ratio of the samples selected is seriously unbalanced, with 169 men entrepreneurs and only 31 women entrepreneurs. This may attribute to the low level of economic development and the lack of an advanced cultural atmosphere, taking men as the main force in entrepreneurial activities.

**FIGURE 4:**
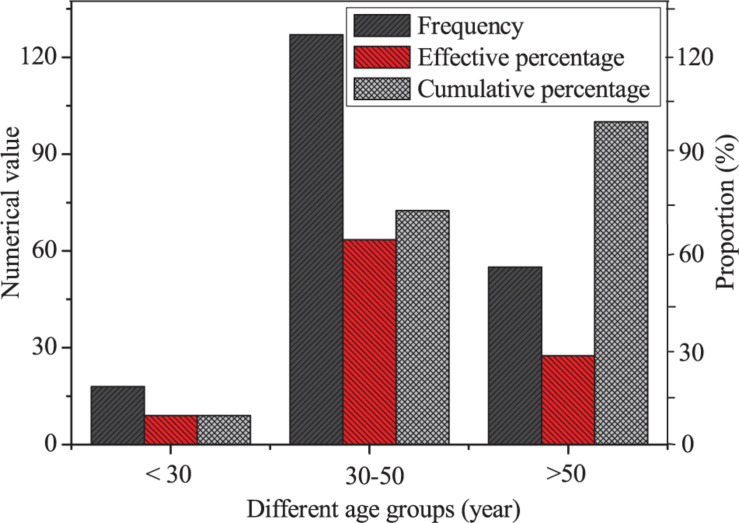
Descriptive statistics of the ages of entrepreneurs.

Second, the descriptive statistics are made on the age of entrepreneurs, and the results are shown in Figure 4.

Figure 4 indicates that there are 18 entrepreneurs under the age of 30 years, accounting for 9% of the total; 127 entrepreneurs are between the age of 30 and 50 years, accounting for 63.5%; and 55 entrepreneurs are over 50 years, accounting for 27.5%. This shows that the age of the entrepreneurs is mainly between 30 and 50 years old.

**FIGURE 5:**
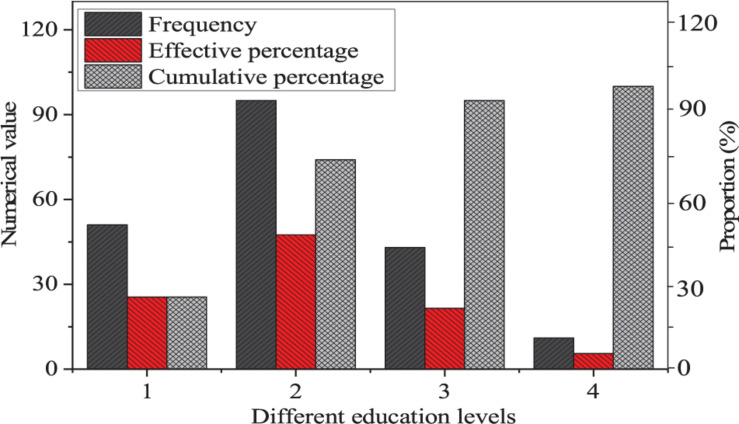
Descriptive statistics of the educational levels of entrepreneurs (1: senior high school or below; 2: junior college; 3: undergraduate; 4: postgraduate and above).

Finally, the descriptive statistics are made on the education level of the entrepreneurs, and the results are shown in Figure 5.

Most of the entrepreneurs are the ones with a junior college education, and the number is 95, accounting for 47.5%, that is, the proportion with a junior college education is the largest, which is consistent with the descriptive statistics of age group.

### Results of the Psychological Characteristics and Entrepreneurial Performance of Entrepreneurs

**FIGURE 6:**
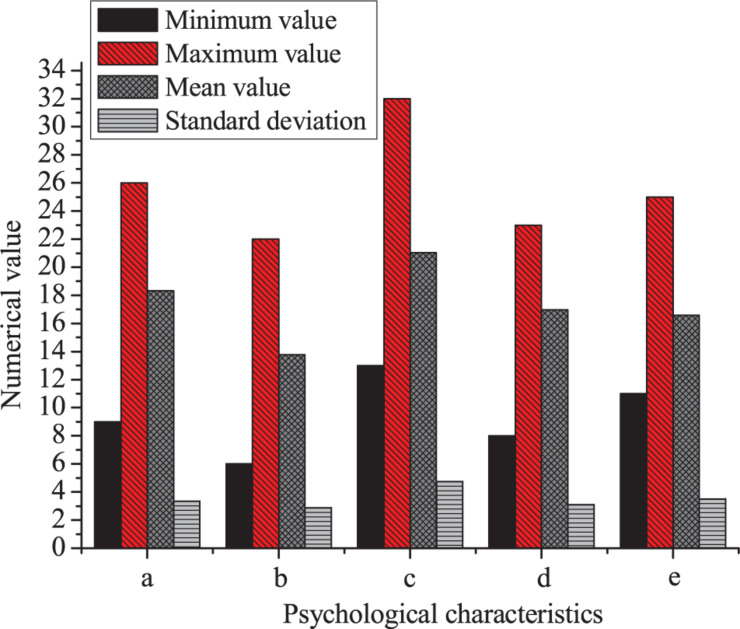
Statistical results of the scores of the psychological characteristics of entrepreneurs (a: success motivation; b: risk bias; c: tolerance; d: control tendency; e: self-confidence).

The results of descriptive statistics on the score of the psychological characteristics of entrepreneurs are shown in Figure 6.

Figure 6 shows that the maximum value of the motivation for success is 26, the minimum is 9, the mean is 18.33, and the SD is 3.356. The maximum value of risk bias is 22, the minimum is 6, the mean is 13.77, and the SD is 2.897. The maximum value of tolerance is 32, the minimum is 13, the mean is 21.03, and the SD is 4.761. The maximum value of control disposition is 23, the minimum is 8, the mean is 16.97, and the SD is 3.115. The maximum value of self-confidence is 25, the minimum is 11, the mean is 16.58, and the SD is 3.526.

**FIGURE 7:**
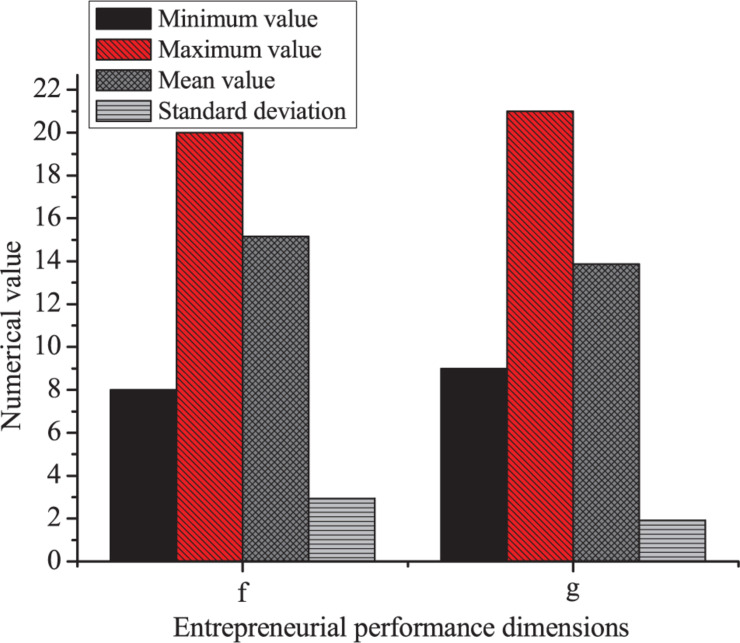
Statistical results of the entrepreneurial performance scale (f: developmental, g: profitable).

The results of descriptive statistics of the performance of entrepreneurs are shown in Figure 7.

Figure 7 indicates the minimum value of developmental performance is 8, the maximum value is 20, the mean is 15.16, and the SD is 2.945. The minimum value of profitable performance is 9, the maximum is 21, the mean is 13.87, and the SD is 1.926.

### Correlation Analysis of the Psychological Characteristics and Entrepreneurial Performance of Entrepreneurs

Hypotheses H1 and H2 are verified by correlation analysis, and the results are shown in Table 5.

**TABLE 5:** Pearson’s correlation coefficient of psychological characteristics and entrepreneurial performance.

	Success motivation	Risk bias	Tolerance	Control tendency	Self-confidence	Development	Profitability
Success motivation	1	0.329*	−0.715**	0.843**	0.732**	0.843**	0.822**
Risk bias	0.329*	1	−0.225	0.226	0.353**	0.211	0.258**
Tolerance	−0.715**	−0.225	1	−0.755**	−0.576	−0.841**	−0.648**
Control tendency	0.843**	0.226	−0.755**	1	0.701**	0.963**	0.886**
Self-confidence	0.732**	0.353**	−0.576**	0.701**	1	0.774**	0.741**
Development	0.843**	0.211	−0.841**	0.963**	0.774**	1	0.675**
Profitability	0.822**	0.258	−0.648**	0.886**	0.741**	0.675**	1

*P < 0.05 and **P < 0.01.

Table 5 shows that the motivation for success has a significant correlation with risk bias on both sides at 0.05 level and has a significant correlation with tolerance, control tendency, self-confidence, development, and profitability at 0.01 level. Risk bias has a significant correlation with self-confidence and profitability; tolerance has a significant correlation with control tendency, development, and profitability; control tendency has a significant correlation with self-confidence and profitability; self-confidence has a significant correlation with development and profitability; development has a significant correlation with profitability, on both sides at 0.01 level.

The figure shows that the success motivation, risk bias, tolerance, control tendency, self-confidence, development, and profitability have significant effects on the psychological characteristics of new entrepreneurs. In the process of entrepreneurship, entrepreneurs have strong self-confidence and a desire for success. Only in this way can entrepreneurs pay attention to risk control, enterprise development, and tolerance of employees, maintaining the growth of business performance and long-term development.

### Analysis of the Influence of the Related Control Variables

**FIGURE 8:**
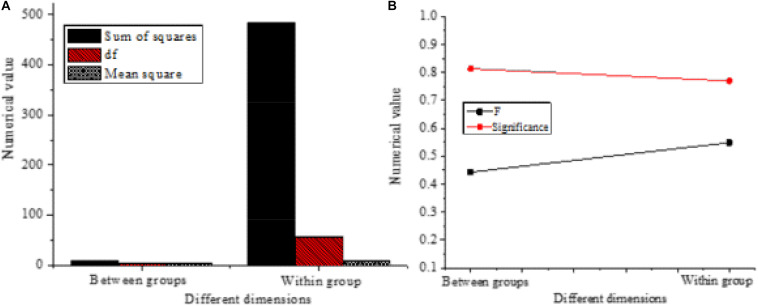
Influence of the genders of entrepreneurs on different variables. **(A)** *t*-test results of mean equation of different variables affected by entrepreneur gender. **(B)** Levene test results of variance equation of different variables affected by entrepreneur gender.

The impact of the genders of entrepreneurs on different variables is analyzed, and the results are shown in Figures 8A,B.

Figure 8A, show that the F values of the overall test are 0.443 (*P* = 0.813 > 0.05) and 0.549 (*P* = 0.769 > 0.05), respectively, which are not significant, indicating that the gender of new entrepreneurs has little impact on entrepreneurial performance.

**FIGURE 9:**
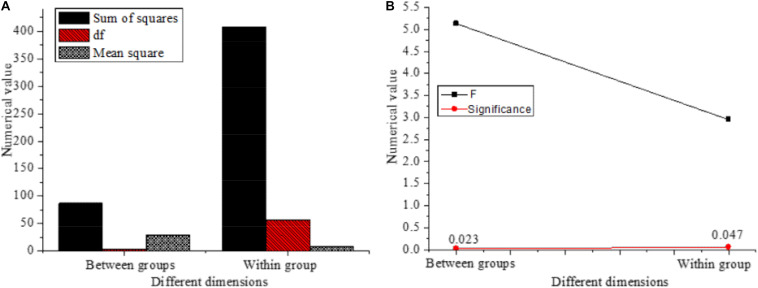
Influence of the ages of entrepreneurs on different variables. **(A)** *t*-test results of the mean equation of different variables affected by entrepreneur age. **(B)** Levene test results of the variance equation of different variables affected by entrepreneur age.

Subsequently, the influence of the ages of entrepreneurs on different variables is analyzed, and the results are shown in Figures 9A,B.

The *F* values of the overall test are 5.131 (*P* = 0.023 < 0.05) and 2.951 (*P* = 0.047 < 0.05), and both are significant, indicating that the ages of entrepreneurs have a great impact on entrepreneurial performance.

**FIGURE 10:**
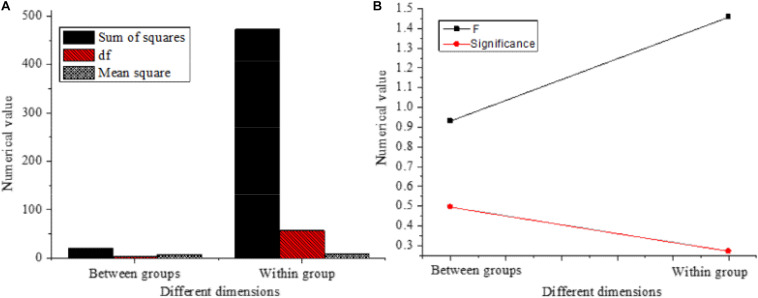
Influence of the education level of entrepreneurs on different variables. **(A)** *t*-test results of the mean equation of different variables affected by entrepreneurs’ education. **(B)** Levene test results of the variance equation of different variables affected by entrepreneurs’ education.

Finally, the influence of the education levels of entrepreneurs on different variables is analyzed, and the results are shown in Figures 10A,B.

The *F* values of the overall test in Figure 10 are 0.931 (*P* = 0.495 > 0.05) and 1.457 (*P* = 0.272 > 0.05), respectively, which are not great, indicating that the education levels of the new entrepreneurs have no significant impact on entrepreneurial performance.

Based on the above, it is concluded that the male-to-female ratio of the entrepreneurs is 169:31, which may be due to the relatively backward cultural atmosphere of entrepreneurship caused by the low level of economic development in the region, making the participants in entrepreneurial activities mainly men; 47.5% of the new entrepreneurs have a college education. Tolerance is scored the highest, which is consistent with the descriptive statistics of ages. That is, older entrepreneurs generally have strong tolerance, which is consistent with the results of Ruiu and Breschi (2019). This study shows that the probability of entrepreneurship of entrepreneurs increases with age (35–44 years old), and the tolerance of entrepreneurs also increases (Ruiu and Breschi, 2019). There is little difference between the maximum scores of developmental performance and profit-seeking performance in entrepreneurial performance of new entrepreneurs, indicating that the new entrepreneurs attach great importance to the development and profit-seeking of enterprises. There is a significant positive correlation between the psychological characteristics of new entrepreneurs and entrepreneurial performance, that is, the psychological characteristics of new entrepreneurs have an impact on the development and profitability of the enterprise, which is consistent with the opinions of Garaika et al. (2019). The results show that the self-efficacy and self-personality of new entrepreneurs have a certain impact on entrepreneurial intention and enterprise efficiency (Garaika et al., 2019).

## Conclusion

Based on the industrial cluster theory in the new era, the research status of the correlation between the psychological characteristics of new entrepreneurs and the performance of new ventures is analyzed, and then the relevant research model is established and two hypotheses are given. Through questionnaire survey and correlation analysis, the hypotheses are verified. It is found that the gender ratio of entrepreneurs in the research object is seriously unbalanced; gender and education have no significant impact on entrepreneurial performance, whereas age has a significant impact on entrepreneurial performance. There is a significant correlation between different types of entrepreneurial psychological characteristics and the two dimensions of entrepreneurial performance.

In the new era of the industrial cluster environment, the cultivation of the psychological characteristics of the new entrepreneurs should be paid attention to; and the self-confidence, the motivation to pursue success, and the ability of risk forecast and control of the entrepreneurs need to be cultivated. The attitude of the leaders toward employees should be tolerant, and the employees are allowed to make mistakes so that their entrepreneurial performance is improved and higher entrepreneurial value is achieved. However, there are some shortcomings in this study. For example, the scope and quantity of the entrepreneur survey are relatively limited, and the dimension analysis of entrepreneurial performance is not enough, which is the content that needs to be analyzed in the next research plan.

## Data Availability Statement

The raw data supporting the conclusions of this article will be made available by the authors, without undue reservation.

## Ethics Statement

The studies involving human participants were reviewed and approved by the Henan University of Technology Ethics Committee. The patients/participants provided their written informed consent to participate in this study. Written informed consent was obtained from the individual(s) for the publication of any potentially identifiable images or data included in this article.

## Author Contributions

The author confirms being the sole contributor of this work and has approved it for publication.

## Conflict of Interest

The author declares that the research was conducted in the absence of any commercial or financial relationships that could be construed as a potential conflict of interest.

## Publisher’s Note

All claims expressed in this article are solely those of the authors and do not necessarily represent those of their affiliated organizations, or those of the publisher, the editors and the reviewers. Any product that may be evaluated in this article, or claim that may be made by its manufacturer, is not guaranteed or endorsed by the publisher.
